# The inconsistency of p-curve: Testing its reliability using the power pose and HPA debates

**DOI:** 10.1371/journal.pone.0305193

**Published:** 2024-07-11

**Authors:** R. Matthew Montoya, Christine Kershaw, Christopher T. Jurgens

**Affiliations:** 1 Department of Psychology, University of Dayton, Dayton, Ohio, United States of America; 2 Department of Psychology, University of Alberta, Edmonton, Alberta, Canada; 3 Department of Psychology, University of Toledo, Toledo, Ohio, United States of America; Villanova University, UNITED STATES

## Abstract

Recent works have called into question whether *p*-curve can reliably assess the presence of "evidential value" within a set of studies. To examine an as-yet unexplored issue, we examined the method used to identify *p*-values for inclusion in a *p*-curve analysis. We developed iterated *p*-curve analysis (IPA), which calculates and *p*-curves every permutation for a set of reported *p*-values, and applied it to the data reported in several published *p*-curve analyses. Specifically, we investigated two phenomena for which *p*-curves have been used to evaluate the presence of evidential value: the power pose and the hypothalamic-pituitary-adrenal (HPA) reactivity debates. The iterated *p*-curve analyses revealed that the *p*-curve method fails to provide reliable estimates or reproducible conclusions. We conclude that *p*-curve should not be used to make conclusions regarding the presence or absence of evidence for a specific phenomenon.

## Introduction

A potential problem when conducting statistical analyses is that researchers may abuse the ambiguity in the data analysis process to ensure a statistically significant finding. This activity has received considerable attention over the decades e.g., [1], and has been called data snooping [2], fiddling [3], bias [4], specification searching [5], post-selection inference [6], researcher degrees of freedom [7], and *p*-hacking [8], among other names. In psychology, the complexity and negative consequences of *p*-hacking were brought to the forefront by two papers authored by Simmons, Simonsohn, and Nelson [7,8].

**Fig 1:**
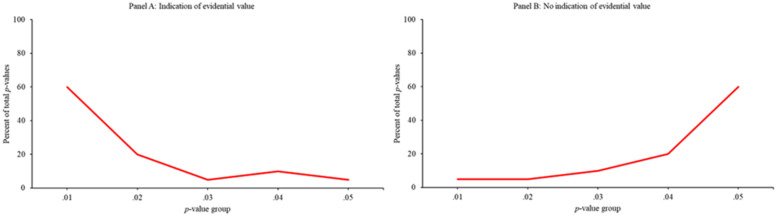
Examples of *p*-curve curves reflective of "evidential value" and "no evidential value". (A) Graph showing evidential value (right skew). (B) Graph showing no evidential value (left skew).

To identify the presence and potential pervasiveness of *p*-hacking, Simonsohn and colleagues [8] developed a new statistical method, *p*-curve. *P*-curve evaluates the distribution of a set of significant *p*-values to determine the presence of "evidential value"—the ability to rule out "selective reporting" (e.g., *p*-hacking) as the sole explanation for a set of findings—within a given set of studies. As discussed in greater depth later, and as illustrated in Fig 1, if the distribution of the set of significant *p*-values extracted from separate studies is skewed to the right (e.g., Fig 1A), evidential value is considered to be present. Alternatively, if the distribution is flat or skewed to the left (e.g., Fig 1B), it suggests the absence of evidential value and/or selective reporting is present [1]. *P*-curve has been the foundation for scores of published articles (e.g., [9,10]) and has been used as a selection bias tool in dozens of meta-analyses (e.g., [11–13]). However, a growing list of investigations has questioned the validity and reliability of *p*-curve. For instance, researchers have noted that the *p*-curve method lacks reliability [14,15] and its statistics are biased when true effect sizes are heterogenous [16,17] and when *p*-hacking has occurred [18], with such research noting that *p*-curve power estimates lack the precision to be informative [19].

In this research, we investigated a different issue with the *p*-curve method that reduces its reliability. Specifically, *p*-curvers are instructed to include a single *p*-value from each study. However, including only a single effect from a set of reported effects may increase the variability in the conclusions drawn from a specific analysis [20]. The goal of this paper was to investigate the impact of selecting a single *p*-value from each study by re-testing several recently published *p*-curve analyses: two that investigated the impact of power posing on the poser’s physiological and psychological state [21,22] and three that investigated the relation between stress and the cortisol response (i.e., hypothalamic-pituitary-adrenal reaction, HPA; [23]).

### *P*-value selection method

*P*-curvers are instructed to include the *p*-value for the hypothesized outcome [8]. Modern researchers, however, tend to include more than one outcome measure or propose a broad hypothesis that can encompass multiple outcome measures. For instance, in a paper included in the *p*-curve analyses of the power posing debate [21,22], Huang et al. hypothesized that power posing—nonverbal displays characterized by expansive and open postures—affects the poser’s "thought and behavior" 24, p. 97. Huang et al. operationalized and tested their hypothesis using a series of assessments, including an abstraction task and a measure of risk orientation. Which *p*-value is the *p*-curver to include, the *p*-value for the abstraction task or for the risk measure? When multiple *p*-values are reported for a stated hypothesis, *p*-curvers are instructed to establish a rule (e.g., "pick the first *p*-value reported, pick the last, pick the highest, pick the lowest") and to follow that rule for selecting *p*-values for all papers in the sample [25]. For Simonsohn and colleagues, which *p*-value is extracted from a particular study is not considered beyond being clearly specified and applying the rule consistently: If there is evidential value, the distribution of *p*-values from a sample of studies produces a right skew. However, we question whether it is of no consequence which *p*-value from a specific study is included in the *p*-curve analysis.

We submit that such an inclusion rule is prone to produce an idiosyncratic sample of *p*-values that can influence the results of the *p*-curve analysis. Most *p*-curvers tend to use the rule "include the first *p*-value reported" (e.g., [26–29]), but other *p*-curvers have used the *p*-value associated with the largest sample [30] or selected a *p*-value at random [31], as discussed in [32]. Moreover, as mentioned, modern researchers often include and report multiple outcome measures. So, which *p*-value do *authors* typically report first? The most significant? The one they *p*-hacked? The most "interesting"? There is no consensus for the order in which authors report the findings from a set of significant outcomes.

The arbitrary order authors use to report their findings, combined with the inclusion rule used by *p*-curvers, results in any significant *p*-value perhaps having an equal chance of inclusion in the *p*-curve analysis. This arbitrariness can have a dramatic impact on the results of a *p*-curve analysis. Consider the hyperstylized example presented in Table 1, in which three studies each present the results from three outcome measures. The three *p*-values arose from three different hypothesized outcome measures, and the order in which they were reported in the results section was arbitrary. One effect from each study is highly significant (*p* = .001) and the other two effects are closer to the *p* < .05 criterion: *p* = .025 and *p* = .049. The *p*-curver is instructed to include only one *p*-value from each study. There are 27 possible permutations of *p*-values that could be included in a *p*-curve analysis. Given both the arbitrary nature of the authors’ reporting order and the researcher’s selected inclusion rule, 3.7% of the time (1 out of 27) a *p*-curver would extract the three *p* = .001 values, and the resulting *p*-curve analysis would produce a right-skewed distribution and support for the conclusion that evidential value is present. Alternatively, if or when authors present their findings in a different order and/or *p*-curvers use a different selection rule, 3.7% of the time the *p*-curver would include the three *p* = .049 values, which would produce a strong left-skewed distribution and the conclusion that the *exact same set* of studies reflects the absence of evidential value (and perhaps strong evidence of *p*-hacking).

**Table 1:** Hypothetical *p*-values reported in an arbitrary order in three different papers.

	Paper #1	Paper #2	Paper #3
Effect #1	*p* = .001	*p* = .001	*p* = .001
Effect #2	*p* = .025	*p* = .025	*p* = .025
Effect #3	*p* = .049	*p* = .049	*p* = .049

The arbitrarily selected *p*-values also impact the estimated power of the sample of studies under consideration. The estimated true power determines the "shape" of the *p*-curve: the larger the true effect, the more right skew that is expected to be evident (both when *p*-hacking is present and absent; see Fig 1 from [8]). Consider the *p*-values from the original Carney et al. paper from the power posing literature [33]. Carney and colleagues examined whether power posing would affect the poser’s physiological processes, behavior, and psychological state. Carney et al. reported, in order, the effect for testosterone (*p* = .045), cortisol (*p* = .009), riskiness (*p* = .049), power feelings (*p* = .003) [33], and thus contributed a single *p*-value to Simmons and Simonsohn’s analysis, the *p* = .045 for testosterone [22]. The *p* = .045 indicates that the power of this study was .52, but if Carney et al. had happened to mention first, say, cortisol, the power from the same study would be estimated at .75. Thus, when a study reports two *p*-values, say, one at .009 and one at .045, the arbitrary selection method not only determines the distribution of *p*-values, but the magnitude of the power estimated from the *p*-curve analysis.

#### Problems with applying existing multivariate methods

Aggregating individual *p*-values across studies without including all reported values (a) removes valuable within-study variance, (b) excludes usable data, and (c) reduces the precision of various summary estimates [34]. A potential solution is to use existing multivariate methods to consider the multiple *p*-values from a given study. One method would be to estimate and include the average of the reported *p*-values [35]. Simonsohn et al., however, note that such a method should not be used: "*P*-curvers should not simultaneously include all of these *p*-values because they must be statistically independent for inference from *p*-curve to be valid" (p. 542) [8]. Alternatively, another proposed method would be to model all reported *p*-values using a multilevel analysis, as analogously used in multilevel meta-analysis (also known as three-level meta-analysis) [35,36], in which multiple *p*-values are statistically "nested" within each paper. However, such an approach would be inconsistent with what *p*-curve is designed to test. *P*-curve examines whether researchers, in essence, "*p*-hacked" their findings to significance. It is unclear whether researchers would *p*-hack *all* their *p*-values or just the *p*-value most crucial to the key hypothesis. Thus, including all *p*-values in the same analysis—many of which may not have been subject to *p*-hacking pressures—would dilute the ability of *p*-curve to detect/reject the presence of evidential value.

In this research, we used a different approach to investigate the effects of *p*-value selection on *p*-curve. Specifically, we examined all reported *p*-values from each study by developing a technique that iterates through all combinations of the relevant reported *p*-values, and then evaluates whether the conclusions differ when different combinations of *p*-values were included in the *p*-curve.

### Purpose of the studies

The goal of this research was to investigate the impact that different combinations of *p*-values reported across and within studies have on the results of a *p*-curve analysis, and thus whether *p*-curve provides reliable or meaningful insight into whether a set of findings has evidential value. Using recent *p*-curve analyses from the (a) power posing phenomenon [21,22] and the (b) HPA axis debate [23], we investigated whether the conclusions from the *p*-curve analyses were dependent on which specific *p*-values were selected for analysis.

#### Iterated *P*-curve Analysis (IPA)

We began with the databases used in the original *p*-curve analyses [21–23]. We then included additional *p*-values that fit Simonsohn and colleagues’ reporting standards (e.g., related to the hypotheses, reported a *p*-value, etc.) [8,25], but ignored the rule that "only the first/second/last *p*-value is to be included".

*IPA script*. We developed a script that executed the *p*-curve app (Version 4.06; available at www.p-curve.com) for every possible permutation of *p*-values in a database. For instance, if each study in a database reported two significant *p*-values, the first iteration would include the first reported *p*-value from all studies, the second iteration would include the second reported *p*-value from the first study and the first *p*-value from all other studies, the third iteration would include the first reported *p*-value from the first study, the second reported *p*-value from the second study and the first *p*-value from all other studies, and so on. The code (in R) for the IPA is available in the (S1, S2 and S4 Files). IPA produces *k*_1_ x *k*_2_ x *k*_n_ *p*-curve permutations, in which *k* is the number of effects from the study, assuming pairs of simple effects count as one effect.

*IPA output*. *P*-curve, and thus IPA, expects the distribution of significant *p*-values to be able to detect whether a set of empirical findings contains evidential value. As noted earlier, Simonsohn et al. [8] state that when there is evidential value, the distribution of significant *p*-values results in a right-skewed distribution. The larger the true effect, the more right skewed the distribution is expected to be. For instance, when the true effect size is moderate (e.g., *d* = 0.60, *N* = 20), there is expected to be about six *p* < .01 for every .04 < *p* < .05, and when there is a large true effect (e.g., *d* = 0.90, *N* = 20), there is expected to be about 18 *p* < .01 for each .04 < *p* < .05 [8]. To test whether the distribution of *p*-values is right skewed, the distribution of significant *p*-values must pass either (a) the *half p-curve test*, which tests whether the distribution of *p*-values below .025 is right-skewed at *p* < .05 (as informed by *z*_Half_), or (b) the combined *half and full p-curve test*, which tests whether the half *p*-curve and the distribution of all *p*-values below .05 are right-skewed at *p* < .10 (as informed by *z*_Half_ and *z*_Full_). This second test ensures the *p*-curve analysis is not underpowered due to excluding *p*-values above .025 [32].

Moreover, *p*-curve, and thus IPA, examines whether the distribution of *p*-values is flat. If the distribution of *p*-values is flat (e.g., if there are a similar number of *p* < .01 as there are .02 < *p* < .03 and .04 < *p* < .05) or not right-skewed (e.g., the number of .025 < *p* < .05 is greater than the number of *p* < .025), it indicates a lack of evidential value, perhaps due to "selective reporting" (e.g., file-drawer problem [37]; *p*-hacking [7]) or a lack of statistical power. For the test for flatness, if the *p*-curve is flatter than expected when the studies are powered at 33%, the *p*-curve analysis indicates a lack of evidential value (as informed by *z*_Full_, *z*_Half_, and *z*_Binomial_).

There is the potential for some permutations to provide clear evidence of evidential value (e.g., evidence of right skew via the half or full *p*-curve test *and* no evidence of flatness) but other permutations to provide evidence for the lack of evidential value (e.g., no evidence of right skew *and* evidence of flatness). In fact, there are four possible outcomes: (a) (evidence of) evidential value, (b) no (evidence of) evidential value, (c) inconclusive, and (d) underpowered. "Evidential value" results from the presence of right skew and the absence of flatness. "No evidential value" is identified when there is no evidence of right skew and the absence of flatness. Whereas an "inconclusive" finding results when there is support for both right skew and flatness, "underpowered" results when there is no support for right skew but there is the presence of flatness. This last category distinguishes a set of findings that is able to rule out selective reporting (i.e., "no evidential value") from a set of findings that lacked the power to detect any evidence.

## Study 1

### The power pose debate

Carney et al.’s seminal article investigated the impact of power posing on the poser’s mental and physiological state. They found that participants who posed using "expansive" versus "contractive" poses (i.e., limbs close to the body) exhibited higher testosterone levels, reduced cortisol levels, greater feelings of power, and made more risky decisions [33]. Their findings sparked a resurgence of interest in the impact of power poses (as first observed by Riskind and Gotay [38]), as evident by scores of power posing studies (for an expansive list, see [39]), one of the most viewed TedTalks [40], and the considerable attention it received in the popular media (e.g., [41–43]).

Confidence in their findings began to erode when a commentary concluded that, based on a *p*-curve analysis, power posing "ought to be treated as hypotheses currently lacking in empirical support" 22, p. 690 In their analysis of the power posing literature, Simmons and Simonsohn tested whether the 33 papers summarized by Carney et al.’s qualitative review [44] provided evidence in support of the power posing effect. Simmons and Simonsohn extracted 31 *p*-values from those papers, and after removing the non-significant *p*-values (specifically, two-tailed *p* > .05), 24 *p*-values were analyzed. They concluded that the distribution of those *p*-values revealed that evidential value was absent because there was no evidence of right skew and there was evidence of flatness [22] (categorized in this research as "underpowered"). In response to those findings, Cuddy et al. conducted a *p*-curve analysis of their own, which included an additional 20 power posing papers, and concluded that evidential value was present in an aggregated *p*-curve across different types of outcome variables, including for subsets of outcomes, specifically, for "feelings of power," and "EASE" (Emotion, Affect, and Self-Evaluation), but not for "non-EASE" outcomes (e.g., pain control, hormones, and risk-taking) [21].

### Data preparation

The first step was to create the databases of relevant *p*-values. The disclosure table for both analyses is presented in the (S3 and S5 Files).

For the Simmons and Simonsohn sample, in addition to the 24 significant *p*-values reported originally [22], inspection of the set of articles produced an addition of 17 significant *p*-values. This sample, however, included 42 *p*-values rather than 41 *p*-values. Whereas *p*-curve excludes non-significant *p*-values from all analyses, non-significant values potentially hold informational value in IPA. For instance, Simmons and Simonsohn did not include a significant *p*-value for Experiment 1 from Welker et al. [45] because the first reported analysis, the omnibus test, was not significant. However, Welker et al. reported a second set of simple effects that would be included in an IPA. Simmons and Simonsohn [22] "non-significant" notation thus provides informational value through its ability to inhibit a set of permutations that include a significant *p*-value. To replicate Simmons and Simonsohn’s analyses, non-significant values were excluded from the reported analyses (except for a non-significant value for the aforementioned Welker et al. paper). Results of the IPA that include non-significant values are reported in the (S4 File).

For the Cuddy et al. *p*-curve (aggregate) analysis, its database contained 42 significant *p*-values [21], and 48 additional significant *p*-values were identified and included. This sample, however, was only computationally feasible after removing two studies ([46, 47], Experiment 2]. We eliminated these two studies because they would have multiplied the number of permutations by 36 and increased the number of permutations to over 2.1 billion. These studies were selected for omission because they both included (a) a large number of *p*-values (both papers reported six usable *p*-values) and (b) *p*-values that, by their exclusion, would contribute to a more conservative test for the presence of evidential value (i.e., the omitted *p*-values would have strongly supported the presence of evidential value; specifically, for Hao et al., the values were *p*s = .0009, .0067, .0092, .0039, .0164, .0285 [47]; for Nair et al., the values were *p*s = .016, .015, .0107, .0053, .0149, .0013 [46]). An IPA that excluded four additional *p*-values identified as “outliers” by Simmons and colleagues [48] produced nearly identical results and is presented in the (S4 File).

All told, for Simmons and Simonsohn’s [22] *p*-curve sample, the IPA script’s iterations of the 42 *p*-values produced 2,592 possible combinations of *p*-values, and for the larger Cuddy et al. [21] sample, the IPA script’s iteration of the 78 *p*-values produced 59,719,680 combinations.

If it is of no consequence which *p*-value is extracted from a specific study, then the results of the Simmons and Simonsohn [22] IPA would reveal little variation across the 2,592 combinations of *p*-values, and the results would align clearly with the *p*-curve analysis presented by Simmons and Simonsohn. The same would hold for the Cuddy et al. [21] *p*-curve analysis, such that it would demonstrate little variation across the 59,719,680 combinations. Alternatively, if it is of consequence which *p*-value is extracted from a particular study, the IPA would produce widely varying combinations of *p*-values, some of which support the presence of evidential value and some of which do not.

## Results

### Simmons and simonsohn sample

**Fig 2:**
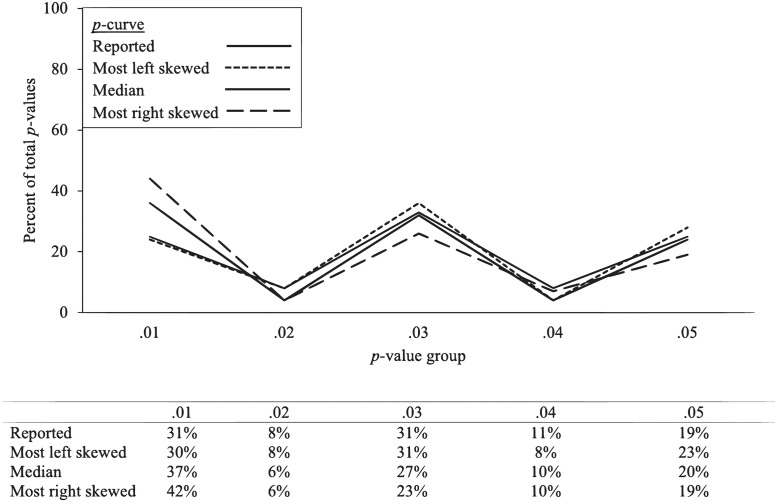
Simmons and Simonsohn (2017) [22] *p*-curve reported results, with most right skewed, most left skewed, and median IPA *p*-curve permutations. Percentage in each *p*-value bin are included in the table below the graph.

Table 2 (top) and Fig 2 presents the results from Simmons and Simonsohn’s [22] analysis and the results from the IPA. To demonstrate the possible range of outcomes of the IPA, we identified the permutation that was associated with the least and most right skew. We identified the extreme permutations by using one of *p*-curve’s estimate of right skew, *z*_Full_. (Other methods to identify the extreme permutations are discussed in the S4 File) The permutations associated with the highest and lowest scores are presented in Table 2 as “most left skewed” and “most right skewed.” We identified the median permutation by finding the median *z*_Full_ value. To further display the variability in the permutations, we also included permutations that were +/- 1SD removed from the median permutation.

**Table 2:** Reported results and the results of the iterated *p*-curve analysis using Simmons and Simonsohn’s [22] sample and Cuddy et al.’s [21] sample.

	Power (90% CI)	Null of no effect (test of right skew)		Null of 33% power (test of flatness)	
		*p*_half_	*p*_full_	*p*_full_	*p*_binomial_
Simmons and Simonsohn [22]					
Reported	5% (5%-14%)	*z* = -1.24, *p* = .107	*z* = 0.31, *p* = .378	*z* = -2.95, *p* = .998	*z* = -2.96, *p* = .001
IPA results					
Most left skewed	5% (5%, 10%)	*z* = -0.46, *p* = .645	*z* = 0.69, *p* = .490	*z* = -3.33, *p* = .999	*z* = -2.59, *p* = .009
-1SD	5% (5%, 18%)	*z* = -1.10, *p* = .271	*z* = -0.04, *p* = .968	*z* = -2.68, *p* = .992	*z* = -2.18, *p* = .029
Median	8% (5%, 27%)	*z* = -1.68, *p* = .092	*z* = -0.77, *p* = .441	*z* = -2.05, *p* = .959	*z* = -1.77, *p* = .076
+1SD	14% (5%, 37%)	*z* = -1.95, *p* = .051	*z* = -1.49, *p* = .136	*z* = -1.51, *p* = .868	*z* = -1.48, *p* = .138
Most right skewed	22% (7%, 46%)	*z* = -3.60, *p* < .001	*z* = -2.31, *p* = .020	*z* = -0.79, *p* = .570	*z* = -1.48, *p* = .138
Cuddy et al. [21]					
Reported	44% (23%-63%)	*z* = -3.71, *p* < .001	*z* = -3.71, *p* < .001	*z* = -0.84, *p* = .799	*z* = -1.75, *p* = .040
IPA results					
Most left skewed	34% (16%-54%)	*z* = -6.43, *p* < .001	*z* = -4.16, *p* < .001	*z* = 0.08, *p* = .063	*z* = -2.01, *p* = .044
-1SD	49% (29%, 67%)	*z* = -7.58, *p* < .001	*z* = -5.54, *p* < .001	*z* = 1.31, *p* = .809	*z* = -1.37, *p* = .170
Median	54% (35%-71%)	*z* = -8.12, *p* < .001	*z* = -6.06, *p* < .001	*z* = 1.80, *p* = .928	*z* = -1.37, *p* = .170
+1SD	59% (40%-74%)	*z* = -8.70, *p* < .001	*z* = -6.58, *p* < .001	*z* = 2.22, *p* = .973	*z* = -1.06, *p* = .289
Most right skewed	73% (57%-84%)	*z* = -10.49, *p* < .001	*z* = -8.31, *p* < .001	*z* = 3.80, *p* > .999	*z* = -0.74, *p* = .459

*Note*. CI = confidence interval. IPA = iterated *p*-curve analysis. *P*-curve results are reported as one-tailed tests, IPA results are reported as two-tailed tests. Left-most, right-most, median, and +/- 1SD were calculated with reference to *z*_Full_.

#### Test of right skew

With respect to the test of whether evidential value is present, the first test, the half *p*-curve, examined whether a significant right skew emerged among *p*-values below *p* = .025. Simmons and Simonsohn reported a non-significant *z*_Half_-score, *z* = -1.24, *p* = .21, which did not provide support for evidential value [22]. For the IPA, it produced a significant median *z*_Half_ score, *z*_Half_ = -1.68, *p* = .04, and a significant most right-skewed permutation, *z*_Half_ = -3.60, *p* < .001. The *z*-score for the -1 SD permutation, *z* = -1.10, *p* = .135, and the most left-skewed, *z* = -0.46, *p* = .322, were not significant, with nearly all permutations less right-skewed than the median not supportive of the presence of evidential value.

We next examined the percent of the permutations that provided support for evidential value using this test. Of the 2,592 permutations, 1,440 (55%) produced a *p*_Half_ below the .05 threshold that supported the presence of evidential value.

The second method to examine support for evidential value is if both the half and full *p*-curves are significant at *p* < .10. Simmons and Simonsohn’s *p*-curve did not support the presence of evidential value using this test (*z*_full_ = 0.31, *p* = .75) [22]. For the IPA, the most right-skewed distribution and +1 SD permutation supported evidential value using this test, but the median and most left-skewed permutation did not. Of the 2,592 permutations, 714 (27%) identified support for evidential value using this test.

#### Test of flatness

The test for the absence of evidential value was examined by a test of whether the full *p*-curve was flatter, at *p* < .05, than the *p*-curve produced assuming the included studies were powered at 33% (or *p* < .10 for both the half and binomial curves). Simmons and Simonsohn’s analysis identified a significant *p*-value (*z*_Full_ = -2.95, *p* = .003), which supported the absence of evidential value [22].

With respect to the IPA results, the most left skewed (*z*_Full_ = -3.33, *p* < .001) and the median permutation (*z*_Full_ = -2.05, *p* = .02) solutions produced significant full *p*-curve estimates, supporting the absence of evidential value. Of the 2,592 permutations, 1,955 (75%) produced a significant *p*-value at .05 for *z*_Full_, indicating that three-fourths of solutions would support the absence of evidential value. With respect to the half/binomial significance test, the *p*-value for all binomial curves was significant (i.e., all *p*_Binomial_ < .10), and given that all *p*_Half_ values were non-significant (in fact, each *p*_Half_ value in every permutation was not significant and are thus not discussed), none of the permutations support the absence of evidential value using this test.

It may appear odd that Simmons and Simonsohn’s solution [22] was not contained within the range of an IPA that included all possible combinations of reported *p*-values. Two studies contribute to this discrepancy: Stepper and Strack [49] and Experiment 1 of Welker et al. [45]. Simmons and Simonsohn reported that Stepper and Strack did not test their hypothesis and thus Simmons and Simonsohn did not include a *p*-value. Instead, Simmons and Simonsohn included the omnibus test in their robustness analysis [22]. The IPA, however, included the *p*-value associated with this omnibus test (*p* = .024) and the other hypothesized *p*-values [49]. Simmons and Simonsohn reported Welker et al. (Experiment 1) as a nonsignificant *attenuated* interaction and therefore included the nonsignificant *p*-value associated with the omnibus test [22]. However, the IPA included the simple effects for the interaction because it is best characterized as a *reversal* interaction. These simple effects included one small, significant *p*-value (*p* < .001) and one nonsignificant *p*-value (*p* = .272) [45]. The inclusion of these effects in the IPA resulted in a distribution of *p*-curves that was less left-skewed than Simmons and Simonsohn’s *p*-curve.

#### Statistical power

Simmons and Simonsohn’s analysis revealed a power estimate of 5% (90% CI: 5%, 14%) [22]. The median IPA produced a larger, but still extremely low, estimate of power, 8% (90% CI: 5%, 27%). The left and right most skewed permutations also produced estimates consistent with extremely low power: for the left-skewed solution, power = 5% (90% CI: 5%, 10%) and right-skewed solution, power = 22% (90% CI: 7%, 46%).

### Cuddy et al. sample

**Fig 3:**
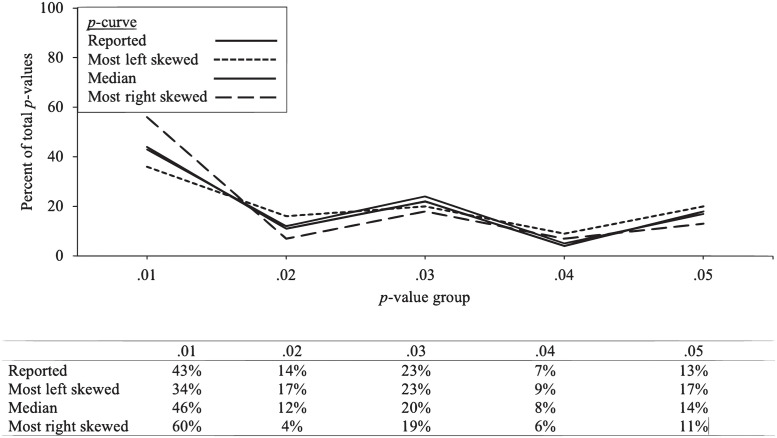
Cuddy et al.’s (2018) [21] *p*-curve reported results, with most right skewed, median, and most left skewed IPA *p*-curve permutations. Percentage in each *p*-value bin are included in the table below each graph.

The results for the Cuddy et al. [21] sample are presented in Fig 3 and Table 2 (bottom).

#### Test of right skew

With respect to the half *p*-curve test, Cuddy et al. reported a significant *z*_Half_-score, *z* = -3.71, *p* < .001, which provided support for evidential value [21]. The IPA revealed that the most left-skewed and most right-skewed permutations were supportive of the presence of evidential value. More specifically, each of the 59,719,680 permutations produced a *p*_Half_ below the .05 threshold that supported the presence of evidential value.

The second method examined if both the half and full *p*-curves are significant at *p* < .10. Cuddy et al.’s *p*-curve supported the presence of evidential value, *z*_Full_ = -3.71, *p* < .001 [21]. With respect to the IPA, both the most right-skewed and most left-skewed distributions identified support for evidential value. Each of the 59,719,680 permutations identified support for evidential value using this test.

#### Test of flatness

The tests for the absence of evidential value examined whether the full *p*-curve is flatter, at *p* < .05, or *p* < .10 for both the half and binomial curves, than the *p*-curve produced assuming the included studies were powered at 33%. Cuddy et al. analysis identified a non-significant *p*-value (*z*_Full_ = -0.84, *p* = .40), which did not support the absence of evidential value [21].

With respect to the results of the IPA, the most left skewed (*z*_Full_ = 0.08, *p* = .532) and most right-skewed permutation (*z*_Full_ = 3.80, *p* > .999) produced non-significant, but positive *z*_Full_ *p*-curve estimates, which do not support the absence of evidential value. Of the 59,719,680 permutations, none supported the absence of evidential value using the *z*_Full_ test. With respect to the half/binomial significant at .10 test, since every *z*_Half_ score was positive, no solution supported the absence of evidential value using this standard.

#### Statistical power

Cuddy et al. reported a power estimate of 44% (90% CI: 23%, 63%) [21]. The most left-skewed permutation exhibited low power (34%; 90% CI: 16%, 54%), but the most right-skewed permutation indicated support for adequate power, 73% (90% CI: 57%, 84%).

### IPA percentages

The Simmons and Simonsohn [22] sample produced conclusions that changed across permutations: 24.57% of permutations provided support for evidential value, 30.97% generated an inconclusive finding, and 44.44% generated an underpowered result. Alternatively, the Cuddy et al. [21] sample provided a more consistent conclusion: 100% of permutations concluded that right-skew was present and did not support the absence of evidential value.

## Study 2

### The HPA debate

Over the past quarter century, various theories have produced competing predictions regarding the relation between trauma and the body’s stress response (i.e., HPA reactivity). On the one hand, it may be that traumatic experiences produce a hyper-reactive stress response. From this perspective, repeated exposure to traumatic events (e.g., death of a loved one) generates a more pronounced stress response (e.g., a more vigilant HPA response) to future threats [50,51]. The body thus becomes “calibrated” to respond to additional stressors with hyper-reactivity. On the other hand, it may be that such traumatic events produce a hypo-reactive stress response [52,53]. This view submits that traumatic experiences do, indeed, initially produce a hyper-reactive response; however, over time, the body compensates for this hyper-reactivity by downregulating its resources, resulting in an under-responsive system. Thus, when exposed to future stress, an HPA hypo-reaction results.

When traditional meta-analyses investigated these competing models, they produced inconsistent conclusions (e.g., [54,55]). To “resolve” the discrepancies between these competing models, Hosseini-Kamkar et al. [23] used *p*-curve to evaluate the evidence for the relation between HPA and trauma. HPA reactivity was exclusively operationalized by cortisol levels, such that greater levels of cortisol were assumed to be associated with a stronger HPA response. Hosseini-Kamkar et al. operationalized stress in one of two ways: (a) traumatic experiences and (b) adverse life experiences (ALE), in which trauma was considered to be exposure to acute adverse events (e.g., death in the family, loss of a job) and adverse life experiences were stressors that persisted throughout development (e.g., poverty, history of childhood maltreatment).

Hosseini-Kamkar et al. completed four *p*-curve analyses: two for the literature that examined the relation between cortisol and trauma (i.e., one *p*-curve of the hyper-reactivity literature and one *p*-curve of the hypo-reactivity literature) and two for the literature that examined the relation between cortisol and adverse life experiences (i.e., one *p*-curve of the hyper-reactivity literature and one *p*-curve of the hypo-reactivity literature) [23]. As summarized in Table 3, Hosseini-Kamkar et al. found different patterns for the relation between HPA and traumatic and adverse life experiences: Whereas the relation between traumatic experiences and hyper-reactivity was inconclusive, the relation between traumatic experiences and hypo-reactivity showed evidential value. The authors concluded that the opposite pattern held for trauma: The literature examining the relation between traumatic events and hyper-reactivity contained evidential value, but the hypo-reactivity literature did not. In short, Hosseini-Kamkar et al. concluded that traumatic events are associated with a lessened cortisol response whereas adverse life experiences are paired with a greater cortisol response.

**Table 3:** Summary of Hosseini-Kamkar et al. [2,3] conclusions.

	Trauma	Adverse life experiences
Hyper-reactivity	Inconclusive	Contained evidential value
Hypo-reactivity	Contained evidential value	No evidential value

### Data preparation

IPAs were conducted to evaluate the reliability of Hoesinni-Kamkar and colleagues’ [23] *p*-curves. The first step was to create a database of the relevant *p*-values. We added 33 *p*-values to the original database of 22 *p*-values for the hyper-reactivity and trauma analysis, 20 *p*-values to the original database of 16 *p*-values for the hypo-reactivity and ALE analysis, and 20 *p*-values to the original database of 15 *p*-values for the hyper-reactivity and ALE analysis. We did not conduct an IPA for the hypo-reactivity and trauma association because the number of *p*-values (*k* = 95) would generate a computationally prohibitive number of permutations (135,444,234,240). The disclosure table for each analysis is presented in the (S3 and S5 Files).

As an important note, Hoesinni-Kamkar et al.’s [23] *p*-curve analyses included several *p*-values that violated Simonsohn et al. inclusion standards [8]. For instance, according to Simonsohn et al., the original authors’, not the *p*-curver’s, stated hypothesis determines which *p*-values should be included in the *p*-curve analysis. Hoesinni-Kamkar et al., however, included several *p*-values that violated this rule. For example, Hoesinni-Kamkar et al. included a *p*-value from Mrug et al. [56] for the correlation between family income and cortisol [23]. However, Mrug et al.’s stated hypothesis was the attenuated interaction of the impact of sleep and gender on cortisol [56]. The three IPAs reported herein reflect Hoesinni-Kamkar et al.’s inclusion standards, in which *p*-values were included that related to cortisol regardless of the original authors’ reported hypothesis [23]. To further investigate whether these violations of Simonsohn et al.’s coding rules affected the IPA results, a second set of IPAs were conducted that met Simonsohn et al.’s *p*-curve inclusion standards. These analyses are reported in the (S4 File).

## Results

**Fig 4:**
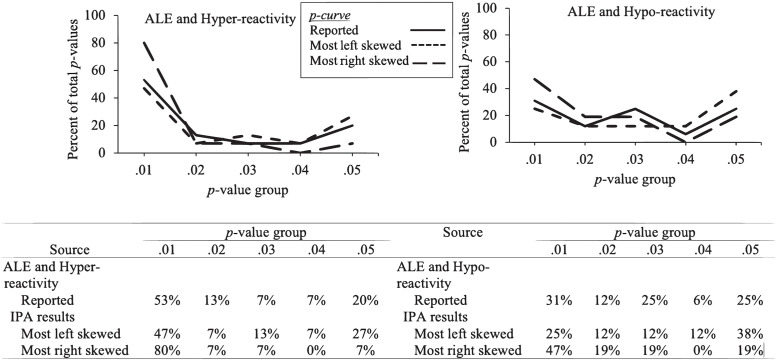
Hoesinni-Kamkar et al. (2021) [23] ALE and hyper-reactivity *p*-curve (left), ALE and hypo-reactivity *p*-curve (right) reported results, with most right skewed and most left skewed IPA *p*-curve permutations. Percentage in each *p*-value bin are included in the table below the graph.

**Fig 5:**
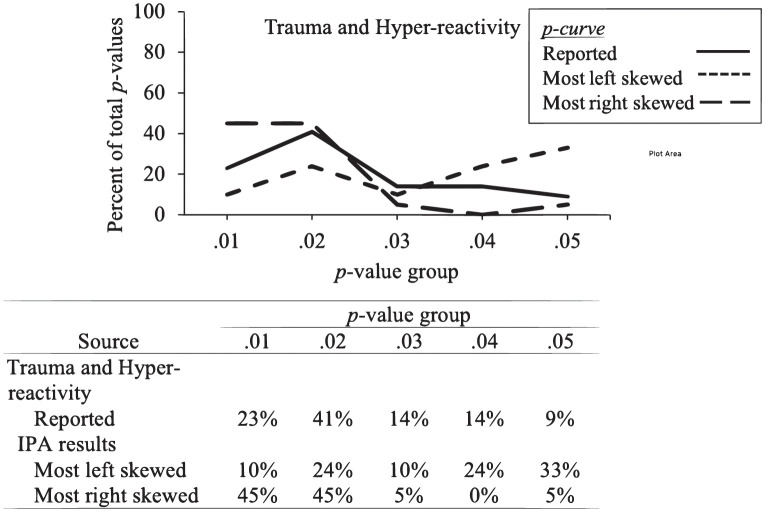
Hoesinni-Kamkar et al. (2021) [23] trauma and hyper-reactivity *p*-curve (left), reported results, with most right skewed and most left skewed IPA *p*-curve permutations. Percentage in each *p*-value bin are included in the table below the graph.

As with Study 1, we began by identifying the possible range of outcomes for the three IPAs by obtaining the “most left skewed” and “most right skewed” permutations. The *z*-scores and *p*-values of the IPA and Hoesinni-Kamkar et al.’s results are presented in Table 4. The ranges, along with the results reported by Hoesinni-Kamkar et al. [23], are presented in Figs 4 and 5.

**Table 4:** Reported results and the results of the iterated *p*-curve analysis using Hoessini-Kamkar et al. [23] sample.

	Power (90% CI)	Null of no effect (test of right skew)		Null of 33% power (test of flatness)	
		*p*_half_	*p*_full_	*p*_full_	*p*_binomial_
Trauma and Hyper-reactivity					
Reported	16% (5%, 42%)	*z* = -1.12, *p* = .132	*z* = 1.61, *p* = .053	*z* = -1.16, *p* = .123	*z* = -0.51, *p* = .305
IPA results					
Most left skewed	5% (5%, 5%)	*z* = 1.18, *p* = .238	*z* = 1.83, *p* = .067	*z* = -4.07, *p* = .999	*z* = -3.55, *p* < .001
Median	17% (5%, 42%)	*z* = -1.30, *p* = .193	*z* = -1.58, *p* = .114	*z* = -1.15, *p* = .749	*z* = -0.50, *p* = .617
Most right skewed	57% (5%, 76%)	*z* = -3.73, *p* < .001	*z* = -4.76, *p* < .001	*z* = 1.57, *p* = .883	*z* = 1.82, *p* = .068
ALE and Hypo-reactivity					
Reported	7% (5%, 29%)	*z* = -1.58, *p* = .057	*z* = -0.40, *p* = .345	*z* = -1.87, *p* = .031	*z* = -2.03, *p* = .021
IPA results					
Most left skewed	5% (5%, 8%)	*z* = -0.65, *p* = .515	*z* = 1.09, *p* = .275	*z* = -3.14, *p* = .998	*z* = -2.53, *p* = .011
Median	9% (5%, 33%)	*z* = -1.49, *p* = .136	*z* = -0.67, *p* = .503	*z* = -1.64, *p* = .898	*z* = -1.01, *p* = .312
Most right skewed	31% (8%, 60%)	*z* = -2.78, *p* = .005	*z* = -2.37, *p* = .017	*z* = -0.15, *p* = .119	*z* = -0.49, *p* = .624
ALE and Hyper-reactivity					
Reported	19% (5%, 50%)	*z* = -2.01, *p* = .022	*z* = -1.56, *p* = .059	*z* = -0.83, *p* = .204	*z* = -0.12, *p* = .452
IPA results					
Most left skewed	8% (5%, 33%)	*z* = -1.39, *p* = .164	*z* = -0.60, *p* = .548	*z* = -1.64, *p* = .898	*z* = -0.67, *p* = .502
Median	41% (14%, 70%)	*z* = -3.09, *p* = .002	*z* = -2.96, *p* = .003	*z* = 0.43, *p* = .332	*z* = 0.46, *p* = .645
Most right skewed	75% (50%, 89%)	*z* = -5.18, *p* < .001	*z* = -5.57, *p* < .001	*z* = 2.68, *p* = .992	*z* = 1.74, *p* = .081

*Note*. CI = confidence interval. IPA = iterated *p*-curve analysis. IPA results are reported as two-tailed tests. *P*-curve results are reported here as they were in the original papers, as one-tailed tests. Left-most, right-most, and median were calculated with reference to *z*_Full_.

### Trauma and hyper-reactivity

Hoesinni-Kamkar et al. reported that their results did not meet either condition for the first and second test of evidential value, *z*_Half_-score = -1.12, *p* = .13; *z*_Full_ = -1.62, *p* = .05 [23]. Alternatively, of the 13,824,000 IPA permutations, 29.37% met the conditions of the first test that support the presence of evidential value, and 53.41% met the conditions of the second test to indicate support of evidential value.

Regarding flatness, Hoesinni-Kamkar et al. reported that their results did not indicate support of the absence of evidential value, *z*_Full_ = -1.16, *p* = .03 [23]. For the IPA, 45.27% of the permutations met the conditions for the first test and 3.89% met the conditions of the second test to indicate support of the absence of evidential value.

#### ALE and hyper-reactivity

Hoesinni-Kamkar et al. reported that their results met the conditions for evidential value, *z*_Half_-score = -2.00, *p* = .02; *z*_Full_ = -1.56, *p* = .05 [23]. Of the 6,912 IPA permutations, 35.56% met the conditions of the first test and 14.95% met the conditions of the second test to indicate support of evidential value.

Hoesinni-Kamkar et al. did not report results for the absence of evidential value [23]. For the IPA, 15.75% of the permutations met the conditions for the first test and 36.37% met the conditions of the second test to indicate support of the absence of evidential value.

#### ALE and hypo-reactivity

Hoesinni-Kamkar et al. reported that their results did not meet the conditions for the first or second test of evidential value, *z*_Half_-score = -1.58, *p* = .05; *z*_Full_-score = -0.40, *p* = .34 [23]. Alternatively, of the 36,864 IPA permutations, 96.35% met the conditions of the first test and 96.30% met the conditions of the second test on support of evidential value.

Regarding flatness, Hoesinni-Kamkar et al. reported that their results indicated support of the absence of evidential value, *z*_Full_ = -1.86, *p* = .03 [23]. For the IPA, 3.65% of the IPA permutations met the conditions for the first test and 3.70% met the conditions of second test that indicate support of the absence of evidential value.

## General discussion

This research used an iterated *p*-curve analysis to evaluate whether *p*-curve would produce different results based on the idiosyncratic sample of *p*-values selected for analysis. Using the power pose and HPA debates as case examples, the analyses revealed that *p*-curve is an unreliable test: The conclusion from a particular *p*-curve test differed due to the specific *p*-values selected for analysis. As summarized in Table 5, although the original papers made definitive claims regarding the presence or absence of evidential value, the IPA revealed that their conclusions may have been affected by the idiosyncratically selected *p*-values. Of the five *p*-curve analyses, the conclusions for four were likely determined by the specific *p*-values that happened to be selected for analysis. More dramatically, the reported conclusions from Hosseini-Kamkar et al.’s [23] trauma and Hyper-reactivity *p*-curve and their ALE and Hypo-reactivity *p*-curve aligned with IPA results that occurred in fewer than 5% of all possible IPA permutations.

**Table 5:** Summary of the original and IPA conclusions.

Source	Original reported conclusion	Number of IPA *p*-values	Number of permutations	IPA results
Simmons & Simonsohn [22]	No evidential value	42	2,592	24.50% evidential value, 30.90% inconclusive, 44.40% underpowered, 0.00% no evidential value
Cuddy et al. [21]	Evidential value	78	59,719,680	100% evidential value, 0.00% inconclusive, 0.00% underpowered, 0.00% no evidential value
Hosseini-Kamkar et al. [23]				
Trauma and Hyper-reactivity	Inconclusive	55	13,824,000	29.37% evidential value, 24.04% inconclusive, 1.32% underpowered, 45.27% no evidential value
ALE and Hyper-reactivity	Evidential value	35	6,912	35.56% evidential value, 34.80% inconclusive, 13.87% underpowered, 15.75% no evidential value
ALE and Hypo-reactivity	No evidential value	36	36,864	96.35% evidential value, 0.00% inconclusive, 0.00% underpowered, 3.65% no evidential value

**Note.** Simmons and Simonsohn’s [22] conclusion of “no evidential value” aligns with our definition of “underpowered.” Hosseini-Kamkar et al.’s conclusion of “inconclusive” [23] aligns with our definition of “underpowered.” ALE = adverse life experiences.

The IPA produced different conclusions for the Simmons and Simonsohn [22] and the Cuddy et al. [21] samples despite both samples testing the power posing literature. One obvious reason is that the two *p*-curve analyses used different samples. Specifically, in the Cuddy et al. sample, there was a higher proportion of *p*-values less than .01 (46% versus 39% for Simmons and Simonsohn’s sample) and fewer *p*-values between .04 and .05 (14% versus 20% for Simmons and Simonsohn’s). Indeed, Simmons et al. [48] have contested that Cuddy et al.’s sample may have contained at least four questionable *p*-values that were statistically unlikely (and possibly outliers) that contributed to their abundance of *p*-values less than .01. Even without those “questionable” *p*-values, however, the *z*_Full_/*z*_Half_ range for the Cuddy et al. sample would continue to be greater than it was for the Simmons and Simonsohn sample, and the IPA would continue to demonstrate considerable variability because each study could have contributed one of several different *p*-values to the *p*-curve analysis. In other words, despite the different sample of studies, the main issue identified by the IPA remains: P-*curve produces results that are dependent on the idiosyncratically selected* p*-values from a sample of studies and it is thus prone to produce inconsistent conclusions*.

The investigation into *p*-curve’s reliability was framed around investigations into the power posing and HPA debates. However, the key conclusion from this paper is that neither the Simmons and Simonsohn [22] nor Cuddy et al. [21] nor Hosseini-Kamkar et al. [23] *p*-curve analysis provides meaningful insight into the existence or absence of the phenomenon.

### Can *P*-curve produce informative results?

Simmons and Simonsohn’s conclusion regarding the presence of evidential value for power posing was definitive, that power poses ought to be “treated as a hypothesis currently lacking in empirical support” [22]. However, the results of the IPA revealed that the statistical method used to produce this conclusion was not reliable. The larger question remains whether *p*-curve (or related analyses) can ever be used to generate an informative conclusion. Simonsohn and colleagues [8] provided the following description for when and how *p*-curve could be used:

We envision that most applications of *p*-curve will involve assessing the evidential value of the core findings put forward in a set of studies, whatever those findings were. For instance, if a *p*-curver were interested in assessing the evidential value of the Top 10 most cited empirical articles published in this journal, her statistical results of interest would be those testing the stated hypotheses in those articles, whatever those hypotheses might be. (p. 543)

Based on the findings of this paper, *p*-curve in these situations would *not* produce informative findings. *P*-curve analyses are subject to produce idiosyncratic results because of the uncertainty regarding which specific *p*-value would be included in the analysis given the possibility of multiple outcome measures and/or a broad hypothesis. In the sample of studies included in the power posing IPAs, for instance, the average number of *p*-values per study from the Simmons and Simonsohn [22] sample was 1.57 (*SD* = 1.00), for the Cuddy et al. [21] sample, it was 1.77 (*SD* = 1.06), and for Hosseini-Kamkar et al.’s [23] sample, collapsing across analyses, it was 2.66 (SD = 1.79), most likely producing the variability in *p*-curve estimates evidenced in this very analysis.

There does, however, remain a narrow set of situations when using *p*-curve would not be subject to the issue raised in this article. *P*-curve might be used when a study meets three conditions: (a) the outcome has been specifically hypothesized (as consistent with Simonsohn et al.’s [8] standards), (b) the hypothesis is identical across each study and paper, and (c) the same outcome measure is used in every study and paper. For example, a *p*-curve analysis not subject to the issues raised herein may be an investigation into the existence of precognitive abilities (e.g., [57]), in which people have an awareness of a future event from which they do not otherwise have the ability to anticipate. As called on by Bem, researchers have repeatedly replicated his test of “retroactive facilitation of recall” (e.g., [58]), in which participants are able to recall expected, but not-yet-practiced words better than those words not expected to be practiced. Such tests all utilized identical methods, hypotheses, and outcome measures. Given a large sample of studies, such a *p*-curve would not be subject the limitations raised in this paper, but may continue to be subject to the various statistical issues that have been identified elsewhere (e.g., [14–19]).

The path to determining the presence or absence of a phenomenon optimally lies in experimental investigation and meta-analyses. For the power posing literature, for instance, an experimental replication using a larger sample failed to reproduce Carney et al.’s [22] original finding [59]. However, a meta-analysis of 48 power posing studies concluded that it was the absence of a contractive pose, rather than an expansive power pose per se, which was responsible for the affective and behavioral changes [39]. Indeed, the power pose literature would be served by an influx of registered replications and/or large-scale registered report studies (e.g., [60,61]).

### Simonsohn et al.’s robustness tests

The robustness tests reported in Simmons and Simonsohn [22], Cuddy et al. [21], and Hosseini-Kamkar et al. [23], each reported conclusions that matched those of the main analysis. The robustness test, as developed by Simonsohn et al. [8], and which take the form of (a) excluding the highest or lowest *p*-value from the sample, (b) using an alternative *p*-value for the included analysis, or (c) replacing one outcome measure for another, does not adequately address the issue raised in this paper.

Specifically, a robustness test that examines a second set of *p*-values from a possible set of 59,719,680 permutations (for the Cuddy et al. database [21]) does not adequately represent the possible range of results that could be produced from a *p*-curve analysis. For the Cuddy et al. sample, for instance, whereas IPA considers 100% of the possible permutations, Simonsohn et al.’s robustness test [8] considered 0.000003% of them. A robustness test would not address the issue even if each paper only reported two *p*-values. For instance, an IPA of Simmons and Simonsohn disclosure table, which included 32 primary *p*-values and 13 robustness *p*-values [22], would produce a set of 768 possible *p*-value permutations, of which only two would be considered using the robustness test (0.26% of the possible permutations).

## Conclusion

Despite its short lifespan, *p*-curve has become a popular meta-analytic tool and lauded as a tool to inform and “settle” popular debates in the academic literature. However, based on the results of five IPAs, two conclusions can be made.

First, *p*-curve, when used as a method to identify evidential value (or as a selection bias tool), cannot produce meaningful and reliable inferences. Our analysis of the broader literature indicates that between 2014 and 2024, over 70 published papers have incorporated a *p*-curve analysis. The findings of this article indicate that selection bias and evidential value claims in such articles should be interpreted with caution. We further caution that IPA should not be used as a “replacement” or “improvement” over *p*-curve. As noted earlier, *p*-curve—and thus IPA—potentially has fundamental problems with the underlying statistical estimation methods, and IPA does not address such concerns. IPA was developed exclusively to demonstrate yet another critical problem with the *p*-curve method.

Second, whereas it may be considered by some an obvious question regarding whether power posing exerts an effect or what the relation is between stress and HPA reactivity, we conclude that *p*-curve cannot meaningfully provide insight into whether these effects are present. We hope these results demonstrate the need for additional research, not only into these phenomena but also meta-analytic quantitative research methods.

## Supporting information

S1 File R Code for IPA. (XLSX)

S2 File Excel file for analyzing IPA data. (R)

S3 File Disclosure table for all analyses. (XLSX)

S4 File Supplementary analyses and instructions for using IPA in R. (DOCX)

S5 File References for articles included in Study 1 and 2 IPA. (DOCX)
