# Supplementary material for: The inconsistency of p-curve: Testing its reliability using the power pose and HPA debates
Source: PLoS One. 2024 Jul 11;19(7):e0305193. doi: 10.1371/journal.pone.0305193 (PMC11239044; doi:10.1371/journal.pone.0305193)
Supplement: S4 File — (DOCX) [file pone.0305193.s004.docx]

**The inconsistency of *p*-curve: Testing its reliability using the power pose and HPA debates**

**S4 File**

**Supplementary Analyses**

**April 1^st^, 2024**

Table of Contents

Page 3 Table S1

*P*-curve information for *p*-curves sorted by *z*_Half_ (right skew) and *z*_Full_ (flatness)

Table 2 presented data that identified those permutations with the maximum/minimum, median, and +/-1 SD *z*_Full_ (right skew) values. However, it is also possible to sort the data by *z*_Half_ (right skew) and *z*_Full_ (flatness). To identify the relevant permutations for *z*_Half_ (right skew), we estimated the range of *z*_Half_ (right skew) and then identified the minimum/maximum values, and calculated the median and +/- 1SD values. To identify the relevant permutations and values for *z*_Full_ (flatness), we replicated the process. This process was applied to both the Simmons and Simonsohn (2017) and Cuddy et al. (2018) samples. Table S1 reports permutations similar to the permutations reported in Table 2.

Page 4 Table S2

*P*-curve information for *p*-curves sorted by *z*_Full_ (right skew) with and without non-significant values

Results of the IPA that include non-significant *p*-values for the Simmons and Simonsohn (2017) sample are presented in Table S2. For this analysis, there were 41 significant values and 23 non-significant values (*k* = 64). Of the 181,440 permutations, 16.88% (30,628) support the presence of evidential value, 44.68% (81,080) are underpowered, 38.43% (69,732) are inconclusive, and 0% support the absence of evidential value. In general, including non-significant *p*-values has a nonmeaningful impact on the median values and the range between the most-skewed permutations. With respect to the percent of permutations, they marginally increase the number of inconclusive permutations while reducing the number that support evidential value.

Page 5 Table S3

*P*-curve and IPA information for Hoessini-Kamkar et al.'s (2021) *p*-curves that met Simonsohn et al.'s *p*-curve inclusion standards

Results of the IPAs that use Simonsohn et al.'s inclusion standards are presented in Table S3. The IPA for trauma and hyperreactivity (*k* = 47) removed or changed 25 *p*-values and added 17 *p*-values. The IPA for ALE and hyperreactivity (*k* = 40) removed or changed 21 *p*-values and added 26 *p*-values. The IPA for ALE and hyporeactivity (*k* = 20) removed or changed 21 *p-*values and adding 5 *p*-values. Although the numbers vary, Table S3 reports permutations with conclusions similar to the conclusions based on the permutations presented in Table 4.

Page 6 Table S4

Summary of the original conclusions and IPA results using Simonsohn et al.'s inclusion standards

Percentage results for the IPA using Simonsohn et al.'s inclusion standards are presented in Table S4. Several of the findings varied from those reported in the main text. Such differences underline the main conclusion of this paper: Even a slight change in the inclusion criteria or reporting standards can drastically affect the results from different *p*-curve analyses of the same set of papers. The most dramatic, and perhaps ironic, change was the ALE and hyporeactive analysis. Hoessini-Kamkar et al. (2021) reported a conclusion of "no evidential value," but the IPA using Hoessini-Kamkar et al.'s (2021) reporting standards indicates that nearly all permutations were consistent with the presence of evidential value. However, adhering strictly to Simonsohn et al.'s standards, the most common result was "no evidential value."

Page 7 Table S5, S6

Summary of the IPA results using Cuddy et al.'s database without "outliers"

In a blog post, Simmons, Nelson, and Simonsohn (2017) submitted that Cuddy et al.'s IPA analyses included four "outliers." We replicated the Cuddy et al. *p*-curve and IPA after removing those four "outliers." Results of the IPA without the four "outliers" are presented in Table S5. Percentage results for the IPA are presented in Table S6. The results are nearly identical to the analyses reported in the article.

Simmons, J. P., Nelson, L. D., & Simonsohn, U. (2017, December 6). [66] *Outliers: Evaluating a new p-curve of power poses*. Data Colada. http://datacolada.org/66

Page 9 Instructions for how to use the IPA R code

Includes the R code's file location and input file formatting instructions.

Page 10 Instructions for using an Excel file to analyze IPA output

An Excel file that identifies the most extreme, median, and +/- 1SD values in IPA output. This file may be used when the number of permutations is fewer than 1,048,576.

Table S1

*Results of the iterated p-curve analysis (IPA) using Simmons and Simonsohn's (2017) and Cuddy et al. (2018) sample sorted by z_Half_ (right skew) and z_Full_ (flatness)*

|  |  |  | Null of no effect  (test of right skew) | |  | Null of 33% power  (test of flatness) | |
| --- | --- | --- | --- | --- | --- | --- | --- |
|  | Power (90% CI) |  | *p*_half_ | *p*_full_ |  | *p*_full_ | *p*_binomial_ |
| Simmons and Simonsohn (2017) | | | | | | | |
| Sorted by *z*_Half_ (right skew) | |  |  |  |  |  |  |
| Most left skewed | 5% (5%, 14%) |  | *z* = -0.13, *p* = .448 | *z* = 0.28, *p* = .611 |  | *z* = -2.97, *p* = .001 | *z* = -2.19, *p* = .014 |
| -1SD | 7% (5%, 24%) |  | *z* = -1.03, *p* = .151 | *z* = -0.54, *p* = .294 |  | *z* = -2.25, *p* = .012 | *z* = -1.77, *p* = .038 |
| Median | 6% (5%, 21%) |  | *z* = -1.93, *p* = .026 | *z* = -0.31, *p* = .378 |  | *z* = -2.44, *p* = .007 | *z* = -2.65, *p* = .004 |
| +1SD | 18% (6%, 41%) |  | *z* = -2.83, *p* = 002 | *z* = -1.93, *p* = .026 |  | *z* = -1.12, *p* = .131 | *z* = 1.48, *p* = .069 |
| Most right skewed | 18% (6%, 41%) |  | *z* = -3.65, *p* < .001 | *z* = -1.90, *p* = .028 |  | *z* = -1.15, *p* = .125 | *z* = 1.88, *p* = .030 |
| Sorted by *z*_Full_ (flatness) | |  |  |  |  |  |  |
| Most left skewed | 5% (5%, 10%) |  | *z* = -0.46, *p* = .322 | *z* = 0.69, *p* = .755 |  | *z* = -3.33, *p* < .001 | *z* = -2.65, *p* = .004 |
| -1SD | 5% (5%, 17%) |  | *z* = -0.89, *p* = .186 | *z* = -0.03, *p* = .488 |  | *z* = -2.70, *p* = .003 | *z* = -2.19, *p* = .014 |
| Median | 8% (5%, 26%) |  | *z* = -1.54, *p* = .061 | *z* = -0.71, *p* = .238 |  | *z* = -2.10, *p* = .017 | *z* = -1.77, *p* = .038 |
| +1SD | 14% (5%, 35%) |  | *z* = -3.12, *p <* .001 | *z* = -1.49, *p* = .068 |  | *z* = -1.51, *p* = .065 | *z* = 1.88, *p* = .030 |
| Most right skewed | 22% (7%, 46%) |  | *z* = -3.60, *p* < .001 | *z* = -2.31, *p* = .010 |  | *z* = -0.79, *p* = .214 | *z* = 1.48, *p* = .069 |
| Cuddy et al. (2018) | | | | | | | |
| Sorted by *z*_Half_ (right skew) | |  |  |  |  |  |  |
| Most left skewed | 43% (24%, 62%) |  | *z* = -5.60, *p* < .001 | *z* = -5.05, *p* < .001 |  | *z* = 0.85, *p* = .803 | *z* = -0.75, *p* = .225 |
| -1SD | 45% (26%, 64%) |  | *z* = -7.38, *p* < .001 | *z* = -5.22, *p* < .001 |  | *z* = 1.02, *p* = .846 | *z* = -1.99, *p* = .023 |
| Median | 52% (32%, 69%) |  | *z* = -8.10, *p* < .001 | *z* = -5.75, *p* < .001 |  | *z* = 1.52, *p* = .936 | *z* = -1.99, *p* = .023 |
| +1SD | 57% (38%, 73%) |  | *z* = -8.82, *p* < .001 | *z* = -6.37, *p* < .001 |  | *z* = 2.04, *p* = .980 | *z* = 1.38, *p* = .083 |
| Most right skewed | 65% (48%, 79%) |  | *z* = -10.95, *p* < .001 | *z* = -7.23, *p* < .001 |  | *z* = 2.88, *p* = .998 | *z* = 1.99, *p* = .023 |
| Sorted by *z*_Full_ (flatness) | |  |  |  |  |  |  |
| Most left skewed | 34% (16%, 54%) |  | *z* = -6.43, *p* < .001 | *z* = -4.16, *p* < .001 |  | *z* = 0.08, *p* = .532 | *z* = -2.01, *p* = .022 |
| -1SD | 49% (29%, 67%) |  | *z* = -7.88, *p* < .001 | *z* = -5.54, *p* < .001 |  | *z* = 1.31, *p* = .905 | *z* = -1.37, *p* = .084 |
| Median | 54% (35%, 71%) |  | *z* = -7.92, *p* < .001 | *z* = -6.05, *p* < .001 |  | *z* = 1.78, *p* = .963 | *z* = -1.37, *p* = .084 |
| +1SD | 59% (40%, 74%) |  | *z* = -8.85, *p* < .001 | *z* = -6.61, *p* < .001 |  | *z* = 2.24, *p* = .987 | *z* = 1.06, *p* = .143 |
| Most right skewed | 73% (57%, 84%) |  | *z* = -10.49, *p* < .001 | *z* = -8.31, *p* < .001 |  | *z* = 3.80, *p* > .999 | *z* = 0.74, *p* = .227 |

*Note*. CI = confidence interval. IPA = iterated *p*-curve analysis. *z*_Half_ (flatness) values are not reported here because each is *p* > .999. One-tailed tests are reported in the table.

Table S2

*Results of the iterated p-curve analysis (IPA) using Simmons and Simonsohn's (2017) sample with and without non-significant values sorted using z_Full_ (right skew)*

|  |  |  | Null of no effect  (test of right skew) | |  | Null of 33% power  (test of flatness) | |
| --- | --- | --- | --- | --- | --- | --- | --- |
|  | Power (90% CI) |  | *p*_half_ | *p*_full_ |  | *p*_full_ | *p*_binomial_ |
| Without non-significant values (as reported in Table 2) | | | |  |  |  |  |
| Most left skewed | 5% (5%, 10%) |  | *z* = -0.46, *p* = .322 | *z* = 0.69, *p* = .755 |  | *z* = -3.33, *p* < .001 | *z* = -2.59, *p* = .005 |
| -1SD | 5% (5%, 18%) |  | *z* = -1.10, *p* = .135 | *z* = -0.04, *p* = .484 |  | *z* = -2.68, *p* = .003 | *z* = -2.18, *p* = .015 |
| Median | 8% (5%, 27%) |  | *z* = -1.68, *p* = .046 | *z* = -0.77, *p* = .220 |  | *z* = -2.05, *p* = .020 | *z* = -1.77, *p* = .038 |
| +1SD | 14% (5%, 37%) |  | *z* = -1.95, *p* = .025 | *z* = -1.49, *p* = .068 |  | *z* = -1.51, *p* = .065 | *z* = 1.48, *p* = .069 |
| Most right skewed | 22% (7%, 46%) |  | *z* = -3.60, *p* < .001 | *z* = -2.31, *p* = .010 |  | *z* = -0.79, *p* = .214 | *z* = 1.48, *p* = .069 |
| With non-significant values | |  |  |  |  |  |  |
| Most left skewed | 5% (5%, 7%) |  | *z* = -0.30, *p* = .038 | *z* = 1.29, *p* = .901 |  | *z* = -3.69, *p* < .001 | *z* = -3.35, *p* < .001 |
| -1SD | 5% (5%, 14%) |  | *z* = -1.26, *p* = .103 | *z* = 0.31, *p* = .621 |  | *z* = -2.94, *p* = .001 | *z* = -2.36, *p* = .009 |
| Median | 7% (5%, 23%) |  | *z* = -1.35, *p* = .088 | *z* = -0.46, *p* = .321 |  | *z* = -2.26, *p* = .011 | *z* = -1.92, *p* = .027 |
| +1SD | 12% (5%, 33%) |  | *z* = -3.44, *p* < .001 | *z* = -1.24, *p* = .108 |  | *z* = -1.69, *p* = .045 | *z* = 2.45, *p* = .007 |
| Most right skewed | 22% (7%, 46%) |  | *z* = -3.60, *p* < .001 | *z* = -2.31, *p* = .010 |  | *z* = -0.79, *p* = .214 | *z* = 1.48, *p* = .069 |

*Note*. CI = confidence interval. IPA = iterated *p*-curve analysis. *z*_Half_ (flatness) values are not reported here because each is *p* > .999. One-tailed tests are reported in the table.

Table S3

*Reported results and the results of the iterated p-curve analysis using Hoessini-Kamkar et al.'s (2021) sample using Simonsohn et al.'s inclusion standards*

|  |  |  | Null of no effect  (test of right skew) | |  | Null of 33% power  (test of flatness) | |
| --- | --- | --- | --- | --- | --- | --- | --- |
|  | Power (90% CI) |  | *p*_half_ | *p*_full_ |  | *p*_full_ | *p*_binomial_ |
| Trauma and Hyper-reactivity | | | | | | |  |
| Reported | 16% (5%, 42%) |  | *z* = -1.12, *p* = .132 | *z* = 1.61, *p* = .053 |  | *z* = -1.16, *p* = .123 | *z* = -0.51, *p* = .305 |
| IPA results |  |  |  |  |  |  |  |
| Most left skewed | 5% (5%, 5%) |  | *z* = 1.10, *p* = .271 | *z* = 2.85, *p* = .004 |  | *z* = -4.87, *p* < .001 | *z* = -2.60, *p* = .009 |
| Median | 12% (5%, 38%) |  | *z* = -1.45, *p* = .147 | *z* = -1.07, *p* = .284 |  | *z* = -1.42, *p* = .078 | *z* = -0.17, *p* = .865 |
| Most right skewed | 48% (21%, 72%) |  | *z* = -2.74, *p* = .006 | *z* = -3.64, *p* < .001 |  | *z* = 0.85, *p* = .197 | *z* = 1.07, *p* = .284 |
| ALE and Hypo-reactivity | | | | | | | |
| Reported | 7% (5%, 29%) |  | *z* = -1.58, *p* = .057 | *z* = -0.40, *p* = .345 |  | *z* = -1.87, *p* = .031 | *z* = -2.03, *p* = .021 |
| IPA results |  |  |  |  |  |  |  |
| Most left skewed | 8% (5%, 34%) |  | *z* = -1.06, *p* = .289 | *z* = -0.49, *p* = .624 |  | *z* = -1.59, *p* = .056 | *z* = -1.05, *p* = .293 |
| Median | 15% (5%, 47%) |  | *z* = -1.47, *p* = .141 | *z* = -1.17, *p* = .242 |  | *z* = -1.01, *p* = .156 | *z* = -0.47, *p* = .638 |
| Most right skewed | 30% (8%, 62%) |  | *z* = -0.86, *p* = .389 | *z* = -2.13, *p* = .033 |  | *z* = -0.17, *p* = .432 | *z* = 0.76, *p* = .447 |
| ALE and Hyper-reactivity | | | | | | | |
| Reported | 19% (5%, 50%) |  | *z* = -2.01, *p* = .022 | *z* = -1.56, *p* = .059 |  | *z* = -0.83, *p* = .204 | *z* = -0.12, *p* = .452 |
| IPA results |  |  |  |  |  |  |  |
| Most left skewed | 8% (5%, 31%) |  | *z* = -2.36, *p* = .018 | *z* = -0.11, *p* = .912 |  | *z* = -2.18, *p* = .015 | *z* = -2.32, *p* = .020 |
| Median | 13% (5%, 39%) |  | *z* = -2.25, *p* = .024 | *z* = -1.13, *p* = .258 |  | *z* = -1.31, *p* = .096 | *z* = -1.34, *p* = .180 |
| Most right skewed | 31% (9%, 60%) |  | *z* = -2.35, *p* = .018 | *z* = -2.52, *p* = .011 |  | *z* = -0.11 *p* = .456 | *z* = 0.33, *p* = .741 |

*Note*. CI = confidence interval. IPA = iterated *p*-curve analysis. IPA results are reported as two-tailed tests. *P*-curve results are reported here as they were in the original papers, as one-tailed tests. Left-most, right-most, and median were calculated with reference to *z*_Full_.

Table S4

*Summary of Hosseini-Kamkar et al.'s (2021) conclusions and IPA results using Simonsohn et al.'s inclusion standards*

| Source | Reported Conclusion | Number of IPA *p*-values | Number of permutations | IPA results |
| --- | --- | --- | --- | --- |
| Trauma and hyper-reactivity | Inconclusive | 22 | 552,960 | 18.91% evidential value,  2.05% inconclusive,  38.38% underpowered,  40.67% no evidential value |
| ALE and hyper-reactivity | Evidential value | 15 | 7,488 | 77.64% evidential value,  16.05% inconclusive,  1.44% underpowered,  4.86% no evidential value |
| ALE and hypo-reactivity | No evidential value | 16 | 48 | 0.00% evidential value,  0.00% inconclusive,  0.00% underpowered,  100% no evidential value |

**Note.** Hosseini-Kamkar et al.'s (2021) conclusion of "inconclusive" aligns with our definition of "underpowered." ALE = adverse life experiences.

Table S5

*Results of the iterated p-curve analysis using Cuddy et al. sample [21] without "outliers" [66]*

|  |  |  | Null of no effect  (test of right skew) | |  | Null of 33% power  (test of flatness) | |
| --- | --- | --- | --- | --- | --- | --- | --- |
|  | Power (90% CI) |  | *p*_half_ | *p*_full_ |  | *p*_full_ | *p*_binomial_ |
| Without "outliers" | 13% (5%, 30%) |  | *z* = -2.58, *p* = .005 | *z* = -1.71, *p* = .044 |  | *z* = -1.91, *p* = .028 | *z* = 0.48, *p* = .314 |
| IPA results |  |  |  |  |  |  |  |
| Most left skewed | 19% (8%, 39%) |  | *z* = -4.46, *p* < .001 | *z* = -2.57, *p* = .005 |  | *z* = -1.22, *p* = .111 | *z* = 0.30, *p* = .380 |
| Median | 38% (20%, 57%) |  | *z* = -6.10, *p* < .001 | *z* = -4.40, *p* < .001 |  | *z* = 0.40, *p* = .657 | *z* = 1.22, *p* = .111 |
| Most right skewed | 59% (41%, 74%) |  | *z* = -8.43, *p* < .001 | *z* = -6.54, *p* < .001 |  | *z* = 2.33, *p* = .990 | *z* = 1.53, *p* = .063 |

*Note*. CI = confidence interval. IPA = iterated *p*-curve analysis. Two-tailed tests are reported in the table. Left-most, right-most, and median were calculated with reference to *z*_Full_.

Table S6

*Summary of Cuddy et al.'s p-curve and IPA without "outliers"*

| Source | Number of  IPA *p*-values | Number of permutations | IPA results |
| --- | --- | --- | --- |
| Cuddy et al. [21] without "outliers" | 74 | 22,394,880 | 100% evidential value,  0.00% inconclusive,  0.00% underpowered,  0.00% no evidential value |

**IPA Script Instructions**

The IPA R code script is available to download as a supplementary file. The IPA script iterates Simonsohn et al.'s (2014a) *p*-curve script (http://www.p-curve.com/) to compute *p*-curves for each permutation of a set of *p*-values. The script uses values from a tab-delimited input file of nested test statistics. The input file uses the following format:

Article Statistic

1 z()=3.45

2 +F(2,210)=4.45

2 +F(2,210)=6.03

2 ++t(88)=2.1

2 ++t(88)=3.8

3 chi2(1)=9.1

3 F(1,100)=3.65

3 r(147)=.246

The first column is composed of study identifiers (e.g., study/paper number) that indicates which test statistics are nested within a study/paper. The second column is composed of test statistics that match the format used in the *p*-curve application (except for *z*-scores, which include a set of empty parentheses). Pairs of simple effects from reversal interactions include a plus (+) or minus (-) sign prior to the paired values. When multiple reversal interactions are nested within a study/paper, pairs of simple effects are distinguished using different signs or by adding additional plus (++) or minus (--) signs.

To execute the script, open the script in R or RStudio, run the full script, and then use the "IPA()" function. The program will prompt the user to specify the input file's format and location, preferred output format (i.e., .RData file or both a .txt and .RData file), and the output file's name.

**Description of how to use Excel file to analyze IPA output**

1. Import the IPA output file into Excel

Save the results of an IPA analysis as a .txt file. The results can be analyzed using the file 'Excel_instructions-analysis.xlsx' available to be downloaded from the Open Science Framework, https://osf.io/5dg8z/?view_only=46228ebef099433c8eee52563fd980a0.

The Excel file includes two worksheets, one labeled 'Instructions' and one labeled 'Raw**_**data'. To add data to be analyzed, open the 'Raw**_**data' worksheet and select cell 'A1'. Then, navigate to the ribbon and select the 'Data' tab. Within this tab, select the option 'From Text', select the IPA output .txt file, and then select 'Get Data'. Use the 'Text Import Wizard' to import the data as delimited data by making sure the delimited option is selected.

2. Reporting results from the analyses

The 'Instructions' worksheet presents output based on several analyses of the raw data. The output 'Calculated values' refers to the median and +/- 1 SD *z*_Full_ (right skew) values that are calculated by adding and subtracting the SD from the median value. These specific values, however, may not exist in the database, so the 'Permutations in the database' contains the closest permutation that matches the 'Calculated values'. The 'Reporting results of the database permutations' presents the values necessary when reporting IPA analyses. Percentages of permutations that support evidential value, its absence, underpowered, or are considered inconclusive, are reported at the bottom of the output.
