# Supplementary material for: The inconsistency of p-curve: Testing its reliability using the power pose and HPA debates
Source: PLoS One. 2024 Jul 11;19(7):e0305193. doi: 10.1371/journal.pone.0305193 (PMC11239044; doi:10.1371/journal.pone.0305193)
Supplement: S5 File — (DOCX) [file pone.0305193.s005.docx]

**The inconsistency of *p*-curve: Testing its reliability using the power pose and HPA debates**

**References**

References marked with an asterisk indicate studies included in the Study 1 IPAs. References marked with a (†) indicate studies included in the Study 2 IPAs.

1. *Allen J, Gervais SJ, Smith JL. Sit big to eat big: the interaction of body posture and body concern on restrained eating. Psychol Women Q. 2013; 37: 325–336. doi: 10.1177/0361684313476477
2. *Arnette SL, Pettijohn TF II. The effects of posture on self-perceived leadership. International Journal of Business and Social Science. 2012; 14: 8–13.
3. *Bohns VK, Wiltermuth SS. It hurts when I do this (or you do that): posture and pain tolerance. J Exp Soc Psychol. 2012; 48: 341–345. doi: 10.1016/j.jesp.2011.05.022
4. *Briñol P, Petty RE, Wagner, B. Body posture effects on self-evaluation: a self-validation approach. Eur J Soc Psychol. 2009; 39: 1053–1064. doi: 10.1002/ejsp.607
5. *Cesario J, McDonald MM. Bodies in context: Power poses as a computation of action possibility. Soc Cogn. 2013; 31: 260–274. doi: 10.1521/soco.2013.31.2.260
6. *Ceunen E, Zaman J, Vlaeyen JWS, Dankaerts W, Van Diest I. Effect of seated trunk posture on eye blink startle and subjective experience: Comparing flexion, neutral upright posture, and extension of spine. PLoS One. 2014; 9: e88482. doi: 10.1371/journal.pone.0088482
7. *Cuddy AJC, Wilmuth C, Yap AJ, Carney DR. Preparatory power posing affects nonverbal presence and job interview performance. J Appl Psychol. 2015; 100: 1286–1295. doi: 10.1037/a0038543
8. *Duffy SE, Feist MI. Power in time: The influence of power posing on metaphoric perspective on time. Lang Cogn. 2017; 9: 637–647. doi: 10.1017/langcog.2016.33
9. *Fischer J, Fischer P, Englich B, Aydin N, Frey D. Empower my decisions: The effects of power gestures on confirmatory information processing. J Exp Soc Psychol. 2011; 47: 1146–1154. doi: 10.1016/j.jesp.2011.06.008
10. *Fuller RC, Montgomery DE. Body posture and religious attitudes. Arch Psychol Relig. 2015; 37: 227–239. doi: 10.1163/15736121-12341310
11. *Garrison KE, Tang D, Schmeichel BJ. Embodying power: A preregistered replication and extension of the power pose effect. Soc Psychol and Personal Sci. 2016; 7: 623–630. doi: 10.1177/1948550616652209
12. *Kozak MN, Roberts T, Patterson KE. She stoops to conquer? How posture interacts with self-objectification and status to impact women's affect and performance. Psychol Women Q. 2014; 38: 414–424. doi: 10.1177/0361684313517865
13. *Kwon J, Kim SY. The effect of posture on stress and self-esteem: Comparing contractive and neutral postures. In Holman R, Cadil J, Cermajova K, Kaderabkova B, Kovar M, Mirvald M, Sevcik M, editors. Proceedings of the 18^th^ International Academic Conference, London. Prague, Czech Republic: International Institute of Social and Economic Sciences (IISES); 2015. pp. 397–404.
14. *Lee EH, Schnall S. The influence of social power on weight perception. J Exp Psychol Gen. 2014; 143: 1719–1725. doi: 10.1037/a0035699
15. *Michalak J, Mischnat J, Teismann T. Sitting posture makes a difference: Embodiment effects on depressive memory bias. Clin Psychol Psychother. 2014; 21: 519–524. doi: 10.1002/cpp.1890
16. *Park LE, Streamer L, Huang L, Galinsky AD. Stand tall, but don’t put your feet up: Universal and culturally-specific effects of expansive postures on power. J Exp Soc Psychol. 2013; 49: 965–971. doi: 10.1016/j.jesp.2013.06.001
17. *Peper E, Booiman A, Lin I, Harvey R. Increase strength and mood with posture. Biofeedback. 2016; 44: 66–72. doi: 10.5298/1081-5937–44.2.04
18. *Riskind JH. Nonverbal expression and the accessibility of life experience memories: A congruence hypothesis. Soc Cogn. 1983; 2: 62–86.
19. *Riskind JH. They stoop to conquer: Guiding and self-regulatory functions of physical posture after success and failure*.* J Pers Soc Psychol. 1984; 47: 479–493. doi: 10.1037/00223514.47.3.479
20. *Roberts T, Arefi-Afshar Y. Not all who stand tall are proud: Gender differences in the proprioceptive effects of upright posture. Cogn Emot. 2007; 21: 714–727. doi: 10.1080/02699930600826432
21. *Rossberg-Gempton I, Poole GD. The effect of open and closed postures on pleasant and unpleasant emotions. Arts Psychother. 1993; 20: 75–82. doi: 10.1016/0197-4556(93)90034-Y
22. *Rotella KN, Richeson JA. Body of guilt: Using embodied cognition to mitigate backlash to reminders of personal and ingroup wrongdoing. J Exp Soc Psychol. 2013; 49: 643–650. doi: 10.1016/j.jesp.2013.02.013
23. *Smith KM, Apicella CL. Winners, losers, and posers: The effect of power poses on testosterone and risk-taking following competition. Horm Behav. 2017; 92: 172–181. doi: 10.1016/j.yhbeh.2016.11.003
24. *Strelan P, Weick M, Vasiljevic M. Power and revenge. Br J Soc Psychol. 2013; 53: 521–540. doi: 10.1111/bjso.12044
25. *Teh P, Lim W M, Ahmed PK, Chan AHS, Loo JMY, Cheong S, Yap W. Does power posing affect gerontechnology adoption among older adults? Behavior & Information Technology. 2017; 36: 33–42. doi: 10.1080/0144929X.2016.1175508
26. *Turan B. Is a submissive posture adaptive when being evaluated negatively? Effects on cortisol reactivity. Neuroendocrinology Letters. 2015; 36: 394–398.
27. *Veenstra L, Schneider IK, Koole SL. Embodied mood regulation: The impact of body posture on mood recovery, negative thoughts, and mood-congruent recall. Cogn Emot. 2017; 31: 1361–1376. doi: 10.1080/02699931.2016.1225003
28. *Wilkes C, Kydd R, Sagar M, Broadbent E. Upright posture improves affect and fatigue in people with depressive symptoms. J Behav Ther Exp Psychiatry. 2017; 54: 143–149. doi: 10.1016/j.jbtep.2016.07.015
29. *Wilson VE, Peper E. The effects of upright and slumped postures on the recall of positive and negative thoughts. Appl Psychophysiol Biofeedback. 2004; 29: 189–195. doi: 10.1023/B:APBI.0000039057.32963.34
30. *Yap AJ, Wazlawek AS, Lucas BJ, Cuddy AJC, Carney DR. The ergonomics of dishonesty: The effect of incidental posture on stealing, cheating, and traffic violations. Psychol Sci. 2013; 24: 2281–2289. doi: 10.1177/0956797613492425
31. *Zabetipour M, Pishghadam R, Ghonsooly B. The impacts of open/closed body positions and postures on learners' moods. Mediterr J Soc Sci. 2015; 6: 643–655. doi: 10.5901/mjss.2015.v6n2s1p643
32. †Afifi T, Davis S, Merrill AF, Coveleski S, Denes A, Afifi W. In the wake of the great recession: economic uncertainty, communication, and biological stress responses in families: in the water of the great recession. Hum Commun Res. 2015; 41: 268–302. doi: 10.1111/hcre.12048
33. †Allwood MA, Gaffey AE, Vergara-Lopez C, Stroud LR. Stress through the mind of the beholder: preliminary differences in child and maternal perceptions of child stress in relation to child cortisol and cardiovascular activity. Stress. 2017; 20: 341–349. doi: 10.1080/10253890.2017.1336617
34. †Aschbacher K, O’Donovan A, Wolkowitz OM, Dhabhar FS, Su Y, Epel E. Good stress, bad stress and oxidative stress: Insights from anticipatory cortisol reactivity. Psychoneuroendocrinology. 2013; *38*: 1698–1708. doi: 10.1016/j.psyneuen.2013.02.004
35. †Andreotti C, Garrand P, Venkatraman SL, Compas BE. (2015). Stress-related changes in attentional bias to social threat in young adults: psychobiological associations with the early family environment. Cognitive Theoretical Research. 2015; 39: 332–342. doi: 10.1007/s10608-014-9659-z
36. †Badanes LS, Watamura SE, Hankin BL. Hypocortisolism as a potential marker of allostaic load in children: Associations with family risk and internalizing disorders. Dev Psychopathol. 2011; 23: 881–896. doi: 10.1017/S095457941100037X
37. †Blankenship SL, Chad-Friedman E, Riggins T, Dougherty LR. Early parenting predicts hippocampal subregion volume via stress reactivity in childhood. Dev Psychobiol. 2019; 61: 125–140. doi: 10.1002/dev.21788
38. †Bosch NM, Riese H, Reijneveld SA, Bakker MP, Verhulst FC, Ormel J, Oldehinkel AJ. Timing matters: long term effects of adversities from prenatal period up to adolescence on adolescents’ cortisol stress response. The TRAILS study. Psychoneuroendocrinology. 2012; 37: 1439–1447. doi: 10.1016/j.psyneun.2012.01.013
39. †Bremner JD, Vythilingam M, Vermetten E, Adil J, Khan S, Nazeer A, Afzal N, McGlashan T, Elzinga B, Anderson GM, Heninger G, Southwick SM, Charnet DS. Cortisol response to a cognitive stress challenge in posttraumatic stress disorder (PTSD) related to childhood abuse. Psychoneuroendocrinology. 2003; 28: 733–750. doi: 10.1016/S0306-4530(02)00067-7
40. †Busse D, Yim IS, Campos B. Social context matters: ethnicity, discrimination and stress reactivity. Psychoneuroendocrinology. 2017; 83: 187–193. doi: 10.1016/j.psyneuen.2017.05.025
41. †Calhoun CD, Helms SW, Heilbron N, Rudolph KD, Hastings PD, Prinstein MJ. Relatoinal victimization, friendship, and adolescents’ hypothalamic-pituitary-adrenal axis responses to an in vivo social stressor. Dev Psychopathol. 2014; 26: 605–618. doi: 10.1017/S0954579414000261
42. †Chiang JJ, Ko A, Bower JE, Taylor SE, Irwin MR, Fuligni AJ. Stress, psychological resources, and HPA and inflammatory reactivity during late adolescence. Developmental Psychopathology. 2019; 31: 699–712. doi: 10.1017/S0954579418000287
43. †Chen G, Kong Y, Deater-Deckard K, Zhang W. Bullying victimization heightens cortisol response to psychosocial stress in Chinese children. J Abnorm Child Psychol. 2018; 46: 1051–1059. doi: 10.1007/s10802-017-036606
44. †Coppens E, Kempke S, Van Wambeke P, Claes S, Morlion B, Luyten P, Van Oudenhove L. Cortisol and subjective stress responses to acute psychosocial stress in fibromyalgia patients and control participants. Psychosom Med. 2018; 80: 317–326.
45. †de Vente W, van Amsterdamn JG, Olff M, Kamphuis JH, Emmelkamp PM. Burnout is associated with reduced parasympathetic activity and reduced HPA axis responsiveness, predominantly in males. Biomed Res Int. 2015; 1–13.
46. †Dietz LJ, Stoyak S, Melham N, Porta G, Matthews KA, Walker Payne M, Brent DA. Cortisol response to social stress in parentally bereaved youth. Biol Psychiatry. 2013; 73: 379–387. doi: 10.1016/j.bipsych.2012.08.016
47. †Doom JR, Cook SH, Sturza J, Kaciroti N, Gearhardt AN, Vazquez DM, Lumeng JC, Miller AL. Family conflict, chaos, and negative life events predict cortisol activity in low-income children. Dev Psychobiol. 2018; 60: 364–379. doi: 10.1002/dev.21602
48. †Ellis BJ, Essex MJ, Boyce WT. Biological sensitivity to context: Empirical explorations of an evolutionary-developmental theory. Developmental Psychopathology. 2005; 17: 303–328. doi: 10.1017/S0954579405050157
49. †Elzinga BM, Spinhoven P, Berretty E, de Jong P, Roelofs K. The role of childhood abuse in HPA-axis reactivity in social anxiety disorder: A pilot study. Biol Psychol. 2010; 83: 396–402. doi: 10.1016/j.psychres.2011.05.046
50. †Fearon RMP, Tomlinson M, Kumsta R, Skeen S, Murray L, Cooper PJ, Morgan B. Poverty, early care and stress reactivity in adolescence: Findings from a prospective, longitudinal study in South Africa. Developmental Psychopathology. 2017; 29: 449–464. doi: 10.1017/S0954579417000104
51. †Flanagan JC, Baker NL, McRae-Clark AL, Brady KT, Moran-Santa Maria MM. Effects of adverse childhood experiences on the association between intranasal oxytocin and social stress reactivity among individuals with cocaine dependence. Psychiatry Res. 2015; 229: 94–100. doi: 10.1016/j.psychres.2015.07.064
52. †Gola H, Engler H, Schauer M, Adenauer H, Riether C, Kolassa S, Elbert T, Kolassa I-T. Victims of rape show increased cortisol responses to trauma reminders: A study in individuals with war-and torture-related PTSD. Psychoneuroendocrinology. 2012; 37: 213–220. doi: 10.1016/j.psyneuen.2011.06.005
53. †Hackman DA, Betancourt LM, Brodsky NL, Hurt H, Farah MJ. Neighborhood disadvantage and adolescent stress reactivity. Front Hum Neurosci. 2012; 6: 1–11. doi: 10.3389/fnhum.2012.00277
54. †Hagan MJ, Roubinov DS, Purdom Marreiro CL, Luecken LJ. Childhood interparental conflict and HPA axis activity in young adulthood: Examining nonlinearrelations: Childhood interparental conflict and the HPA axis. Dev Psychobiol. 2014; 56: 871–880. doi: 10.1002/dev.21157
55. †Harkness KL, Stewart JG, Wynne-Edwards KE. Cortisol reactivity to social stress in adolescents: Role of depression severity and child maltreatment. Psychoneuroendocrinology. 2011; 36: 173–181. doi: 10.1016/j.psyneuen.2010.07.006
56. †Hibel LC, Granger DA, Blair C, Cox MJ, Family Life Project Key Investigators. Maternal sensitivity buffers the adrenocortical implications of intimate partner violence exposure during early childhood. Developmental Psychopathology. 2011; 23: 689–701. doi: 10.1017/S0954579411000010
57. †Ivanov I, Yehuda R, Greenblatt E, Davidow J, Makotkine I, Alfi L, Newcorn JH. The effect of trauma on stress reactivity in aggressive youth. Psychiatry Res. 2011; 189: 396–402. doi: 10.1016/j.psychres.2011.05.046
58. †Jaffee SR, McFarquhar T, Stevens S, Ouellet-Morin I, Melhuish E, Belsky J. Interactive effects of early and recent exposure to stressful contexts on cortisol reactivity in middle childhood. J Child Psychol Psychiatry. 2015; 56: 138–146. doi: 10.1111/jcpp.12287
59. †Kern S, Laurent HK. Childhood abuse predicts affective symptoms via HPA reactivity during mother-infant stress. Psychoneuroendocrinology. 2019; 107: 19–25. doi: 10.1016/j.psyneuen.2014.10.027
60. †Koss KJ, George MRW, Davies PT, Cicchetti D, Cummings EM, Sturge-Apple ML. Patterns of children’s adrenocortical reactivity to interparental conflict and associations with child adjustment: A growth mixture modeling approach. Develop Psychol. 2013; 49: 317–326. doi: 10.1037/a0028246
61. †Kuhlman KR, Vargas I, Geiss EG, Lopez-Duran NL. Age of trauma onset and HPA axis dysregulation among trauma-exposed youth: Age of first trauma and HPA axis functioning. J Trauma Stress. 2015; 28: 572–579. doi: 10.1002/jts.22054
62. †Laceulle O, Nederhof E, Aken M, Ormel J.. Adversity-driven changes in hypothalamic-pituitary-adrenal axis functioning during adolescence. The TRIALS study. Psychoneuroendocrinology. 2017; 85: 49–55. doi: 10.1016/j.psyneuen.2017.08.002
63. †Lucas-Thompson RG, Lunkenheimer ES, Granger DA. Adolescent conflict appraisals moderate the link between marital conflict and physiological stress reactivity. J Res Adolesc. 2017; 27: 173–188. doi: 10.1111/jora.12264
64. †Luecken LJ. Childhood attachment and loss experiences affect adult cardiovascular and cortisol function. Psychosom Med. 1998; 60: 765–772. doi: 10.1016/S0022-3999(00)00151.3
65. †Luecken LJ, Appelhans BM. Early parental loss and salivary cortisol in young adulthood: The moderating role of family environment. Developmental Psychopathology. 2006; 18: 295–308. doi: 10.1017/S09545794060160
66. †McFadyen-Ketchum LS, Hurwich-Reiss E, Stiles AA, Mendoza MM, Badanes LS, Dmitrieva J, Watamura SE. Self-regulation and economic stress in children of Hispanic immigrants and their peers: Better regulation at a cost? Early Educational Development. 2016; 27: 914–931. doi: 10.1080/10409289.2015.1036345
67. †Mezuk B, Lexima E, Kalesnikava V, Fleming J, Montgomery J, Tuktur W, Winston J, Perrin PB, Green T, Wheeler DC. Stress-reactivity as a contributor to racial and socioeconomic disparities: Rationale and baseline results from the Richmond Stress and Sugar Study. Psychosom Med. 2020; 82: 658–668. doi: 10.1097/psy.0000000000000830
68. †Mielock AS, Morris MC, Rao U. Patterns of cortisol and alpha-amylase reactivity to psychosocial stress in maltreated women. J Aff Disord. 2017; 209: 46–52. doi: 10.1016/j.jad.2016.11.009
69. †Neupert SD, Miller LMA, Lachman ME. Physiological reactivity to cognitive stressors: Variations by age and socioeconomic status. Int J Aging Hum Dev. 2006; 62: 221–235. doi: 10.2190/17DU-21AA-5HUK-7UFG
70. †Obasi EM, Shirtcliff EA, Cavanagh L, Ratliff KL, Pittman DM, Brooks JJ. Hypothalamic-pituitary-adrenal reactivity to acute stress: An investigation into the roles of perceived stress and family resources. Prev Sci. 2017; 18: 923–931. doi: 10.1007/s11121-017-0759-3
71. †Ouellet-Morin I, Boivin M, Dionne G, Lupien SJ, Arsenault L, Barr RG, Perusse D, Tremblay RE. Variations in heritability of cortisol reactivity to stress as a function of early familiat adversity among 19-month-old twins. Arch Gen Psychiatry. 2008; 65: 211–219. doi: 10.1001/archgenpsychiatry.2007.27.
72. †Pesonen A-K, Räikkönen K, Feldt K, Heinonen K, Osmond C, Phillips DIW, Barker DJP, Eriksson JG, Kajantie E. Childhood separation experience predicts HPA axis hormonal responses in late adulthood: A natural experiment of World War II. Psychoneuroendocrinology. 2010; 35: 758–767. doi: 10.1016/j.psyneuen.2009.10.017
73. †Quas JA, Yim IS, Oberlander TF, Nordstokke D, Essex MJ, Armstrong JM, Bush N, Obradovic J, Boyce WT. The symphonic structure of childhood stress reactivity: Patterns, of sympathetic, parasympathetic, and adrenocortical responses to psychological challenge. Developmental Psychopathology. 2014; 26: 963–982. doi: 10.1017/S0954579414000480
74. †Raffington L, Prindle J, Keresztes A, Binder J, Heim C, Shing Y L. Blunted cortisol stress reactivity in low-income children relates to lower memory function. Psychoneuroendocrinology. 2018; 90: 110–121. doi: 10.1016/j.psyneuen.2018.02.002
75. †Rao U, Hammen C, Ortiz LR, Chen L-A, Poland RE. Effects of early and recent adverse experiences on adrenal response to psychosocial stress in depressed adolescents. Biol Psychiatry. 2008; 64: 521–526. doi: 10.1016/j.biopsyvh.2008.05.012
76. †Shalev A, Porta G, Biernesser C, Zelazny J, Walker-Payne M, Melhem N, Brent D. Cortisol response to stress as a predictor for suicidal ideation in youth. J Affect Disord. 2019; 257: 10–16. doi: 10.1016.j.jad.2019.06.053
77. †Tackett JL, Herzhoff K, Smack AJ, Reardon KW, Adam EK. Does socioeconomic status mediate racial differences in the cortisol response in middle childhood? Health Psychol. 2017; 36: 662–672. doi: 10.1037/hea0000480
78. †van der Hal-Van Raalte EAM, Bakermans-Kranenburg MJ, van IJzendoorn MH. (2008). Diurnal cortisol patterns and stress reactivity in child Holocaust survivors reaching old age. Aging Ment Health. 2008; 12: 630–638. doi: 10.1080/13607860802343134
79. †Wan C, Couture-Lalande M-E, Lebel S, Bielajew C. The role of stressful life events on the cortisol reactivity patterns of breast cancer survivors. Psychol Health. 2017; 1–17. doi: 10.1080/08870446.2017.1346194
80. †Wirtz PH, Ehlert U, Kottwitz M U, La Marca R, Semmer NK. Occupational role stress is associated with higher cortisol reactivity to acute stress. J Occup Health Psychol. 2013; 18: 121–131. doi: 10.1037/a0031802
81. †Zwolinski J. Biopsychosocial responses to social rejection in targets of relational aggression. Biolo Psychol. 2008; 79: 260–267. doi: 10.1016/j.biopsycho.2008.06.006
